# Adult metanephric adenoma presumed to be all benign? A clinical perspective

**DOI:** 10.1186/s12885-015-1211-3

**Published:** 2015-04-25

**Authors:** Gang Li, Yuhong Tang, Renya Zhang, Hualin Song, Shumin Zhang, Yuanjie Niu

**Affiliations:** 1 Department of Urology, The second Hospital of Tianjin Medical University, Tianjin Institute of Urology, Tianjin, 300211 China; 2 Hebei North University, Laboratory Medicine College, Zhangjiakou, 075000 China; 3 Department of Pathology, Affiliated Hospital of Jining Medical University, Jining, China

**Keywords:** Metanephric adenoma, MA, Benign tumour, MA subtypes, Clinicopathological characteristic

## Abstract

**Background:**

In most documented literature, metanephric adenoma (MA) is described as a benign tumour. Nevertheless, the nature of MA remains unclear and the clinical criteria of different MA subtypes are not well established. In the present study, we investigated the clinicopathological characteristics of MA, especially those of the uncommon histological subtypes.

**Methods:**

A cohort study was performed on 18 patients with pathologically proven MA in our institute from January 2004 to June 2014. The patients’ clinicopathological and radiological data were retrospectively analysed and evaluated with an emphasis on the corresponding subtypes.

**Results:**

The patient population had a female: male ratio of 1:1 and mean age of 50 years (range, 18–66 years). The mean tumour size was 3.9 cm (range, 1.4–9.0 cm). There were no pathognomonic radiological features that posed a challenge for a preoperative diagnosis of MA. Fourteen patients underwent radical nephrectomy, and the other four underwent partial nephrectomy. Three histological subtypes were observed: classic MA (n = 10), malignant MA (n = 2), and composite MA with coexistence of different malignant components (n = 6). Despite the presence of atypical histological features and malignant components among the patients, only one patient developed distant metastasis (median postoperative follow-up, 56 months; range, 30–86 months).

**Conclusions:**

MAs are a heterogeneous group of neoplasms with different biological characteristics. The correct identification of this entity and its subtypes would facilitate stratification of optimal management protocols and accurate assessment of the prognosis.

## Background

The term ‘metanephric adenoma’ (MA) was originally described by Bove in 1979 and is known to be associated with Wilms’ tumour [1]. To date, fewer than 200 cases of MA have been reported worldwide in the English-language literature. In most documented literature, MA is characterised as a rare benign tumour of the kidney that accounts for approximately 0.2% of adult renal epithelial neoplasms. It generally occurs in adults and has an excellent prognosis. Nevertheless, the detailed nature of MA remains unclear. Several reports have suggested that a small subset of these tumours has atypical histological features or even an exponential growth pattern [2], and the capacity for MA to become malignant has been reported [3]. However, the clinical criteria of different MA subtypes are not well established. In the present study, we investigated different MA subtypes and aimed to establish clinical criteria that will facilitate more accurate therapy planning by using pathological findings as the gold standard. Limited clinical data on MA are available in the English-language literature. To the best of our knowledge, this is the largest clinical series to date focusing on clinical and pathological subtype analysis of MA.

## Methods

### Study design and patient selection

This retrospective observational cohort study was approved by the Institutional Review Board of the Second Hospital of Tianjin Medical University. The study was approved by all patients and written informed consents were obtained from all patients to publish their clinical details and images. The medical records of 18 patients with pathologically proven MA were retrieved from the archival files and retrospectively analysed in our institute from January 2004 to June 2014. All pathologic specimens were acquired after surgery, and none were diagnosed by biopsy. Preoperative abdominal ultrasound and computed tomography (CT) examinations were performed in all cases; magnetic resonance imaging was performed in only three cases. The patients’ demographic characteristics, clinical presentation, radiological characteristics (tumour diameter, location, CT value, and growth and enhancement patterns), histological findings, and perioperative and follow-up data were recorded. Details of the patient’s clinicodemographic characteristics and CT findings are listed in Table 1. Abdominal biphasic CT scans and three-phase contrast-enhanced CT scans were performed in all cases. Data on calcification, tumour-spreading patterns, lymphadenopathy, and enhancement patterns (homogeneous, heterogeneous) were recorded and retrospectively analysed. All patients were treated surgically; 14 underwent radical nephrectomy, and four underwent partial nephrectomy. The tumour grade was assigned according to the World Health Organization grading system. All pathological diagnoses were determined by at least two urological pathologists. In inconclusive cases, the final diagnosis was determined after consultation with senior pathologists. No patients received any adjuvant therapeutic modalities. The median follow-up period was 56 months (range, 30–86 months). The therapeutic modalities, pathological findings, and follow-up data are detailed in Table 1. The types of surgical interventions, complications, postoperative management, and survival results were all retrospectively analysed.

**Table 1:** **The clinical, CT and pathological characteristics of MA**

NO.	Sex/Age (years)	Tumor size (cm)	Unenhanced/enhanced attenuation(Hu)	Treatment modality	Pathology diagnosis	Recurrence or metastasis
1	F/48	2	37/61	NSS	MA	NO
2	F/65	3	30/87	RN	MA	NO
3	M/62	6.2	9/12	RN	MA	NO
4	M/45	4	21/42	RN	MA, PT	NO
5	F/33	2	20/25	NSS	MA	NO
6	F/64	4.5	30/46	RN	MA	NO
7	F/53	6.5	36/58	RN	MA, PT	NO
8	M/64	6	45/71	RN	MA	NO
9	F/38	5	46/107	RN	MA	NO
10	M/65	2.7	43/51	NSS	MA, AC	NO
11	F/50	9	20/58	RN	MA, CCC,	NO
12	M/50	4.3	25/53	RN	MA	NO
13	F/38	3.5	25/30	NSS	Malignant	NO
14	M/33	3.5	24/58	RN	MA	NO
15	F/47	4.7	33/76	RN	MA,OC	NO
16	M/43	4	26/64	RN	Malignant	M
17	M/51	3	28/34	RN	MA	NO
18	M/18	5.3	32/46	RN	MA, PT	NO

F: female, M: male, RN: radical nephrectomy, NSS: nephron sparing surgery, MA: Metanephric adenomas, CCC:chromophobe cell carcinoma, OC: Oncocytic carcinoma, PT: Papillary tumor, AC: adenocarcinoma, M: metastasis.

### Statistical analysis

The chi-squared test was used for categorical variables. All reported nonparametric p-values are two-sided, and statistical significance was set at p < 0.05. Ratios were compared between the two groups using T tests. All data were analysed using SPSS, version 17 (SPSS Inc., Chicago, IL, USA).

## Results

### Clinical data and surgical treatment

In nine patients, the tumours were incidentally detected on imaging studies performed for unrelated clinical presentations. Gross haematuria was found in five patients, and loin pain was present in four patients. For small tumours (<4 cm), the choice of surgical approach depended on the patient’s compliance and the attending urologist’s individual preference. In complicated circumstances, such as tumour localisation in the central part of the kidney or the presence of an entophytic tumour, partial nephrectomy is very difficult. Surgical parameters including tumour stage, tumour size, operating time, warm ischaemia time, and complications were documented. In the partial nephrectomy group, the mean preoperative tumour size was 2.5 cm (range, 1.4–3.5 cm), and the clinical stage was T1a. All patients in the partial nephrectomy group underwent pedicle clamping, and the mean (± standard deviation) warm ischaemia time was 26 ± 6 min. All surgical margins were negative. In the radical nephrectomy group, the mean preoperative tumour size was 5.2 cm (range, 3.5–9.0 cm), and the clinical stages were T1a (n = 13) and T1b (n = 1). One patient underwent resection of an enlarged lymph node with a pathologically proven inflammatory reaction. All patients tolerated the surgery well and had an unremarkable postoperative recovery.

### Radiological findings

**Figure 1:**
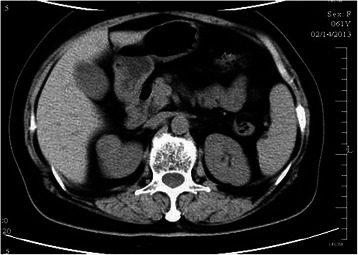
CT showing the presence of homogeneity and well-defined solid renal masses.

**Figure 2:**
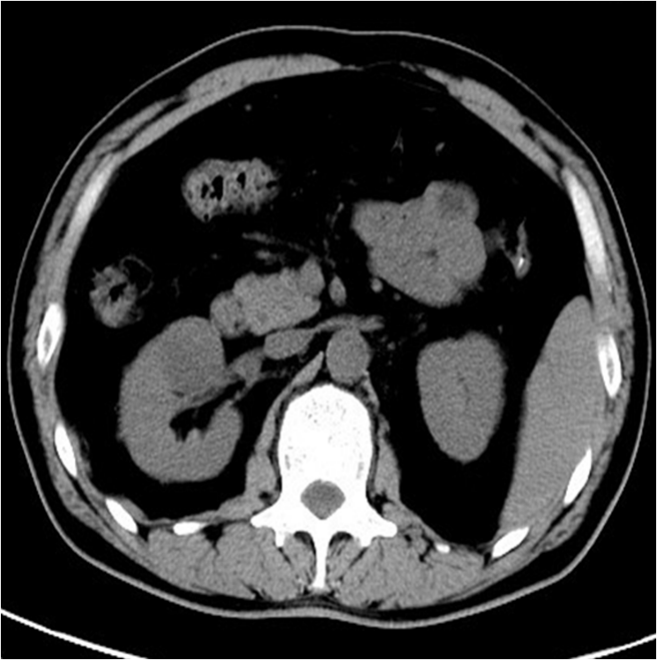
CT showing a heterogeneous or centrally located low-attenuation mass.

**Figure 3:**
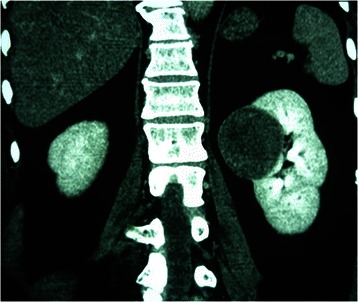
Contrast-enhanced CT image revealed heterogeneous and varying degrees of enhancement.

Ten tumours were found on the right side and eight were found on the left side. Seven tumours were found in the upper pole of the kidney, five in the middle pole, and six in the lower pole. The most common imaging characteristic on unenhanced abdominal CT was the presence of homogeneous, well-defined solid renal masses (n = 15, 83.3%) (Figure 1); the least common was the presence of heterogeneous or centrally located low-attenuation masses (n = 3, 16.7%) (Figure 2). Contrast-enhanced CT revealed heterogeneity and varying degrees of enhancement in 16 (88.9%) tumours (Figure 3), while 2 (11.2%) tumours did not exhibit increased attenuation. Scattered calcification and an enlarged lymph node were found in only one patient (5.6%).

### Pathological findings

**Figure 4:**
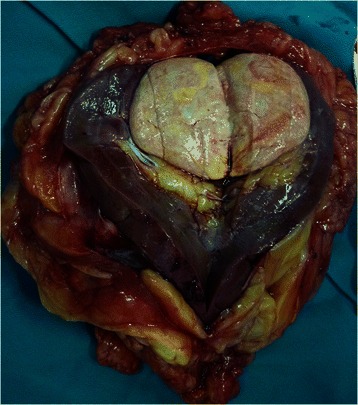
Macroscopically, MA revealed a homogenous tan or yellowish-white colour cut surface and the encapsulated tumour generally formed well-circumscribed mass.

**Figure 5:**
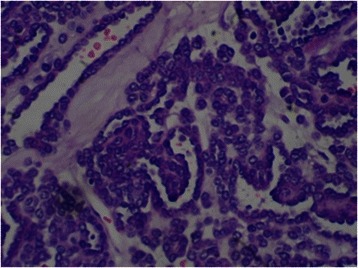
Microscopically,the tumour was composed of variable proportions of cells proliferated with formation of small glomeruloid bodies.

**Figure 6:**
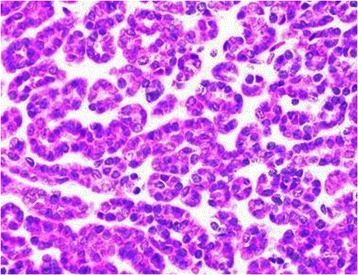
Tumours cells had uniformly small and indistinct nucleoli, and scanty cytoplasm.

**Figure 7:**
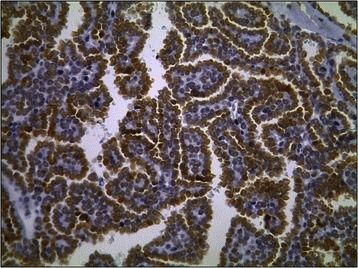
Immunohistochemical staining revealed most tumor cells were positive expression of WT-1 (original magnification, ×200).

**Figure 8:**
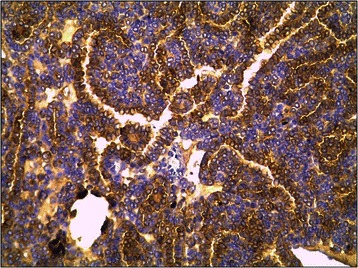
Immunohistochemical staining of tumor cells were positive for CD57 (original magnification, ×200).

**Figure 9:**
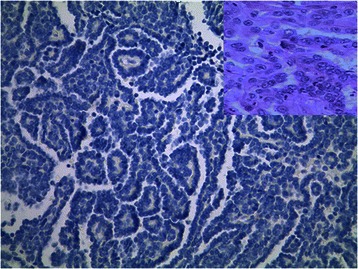
Tumour cells were composed of epithelial elements and lack of typical architecture of tubules and glomeruloid bodies (original magnification, ×100), atypia and mitotic activity were present (original magnification, ×200).

Macroscopically, the MAs ranged in size from 1.4 to 9.0 cm (mean, 4.5 cm). The cut surface was a homogenous tan or yellowish-white colour, and the tumour was an encapsulated, generally well-circumscribed mass (Figure 4). Microscopically, the MAs comprised variable proportions of proliferating cells forming small glomeruloid bodies (Figure 5). The tumour cells had uniformly small and indistinct nucleoli and scanty cytoplasm (Figure 6). Immunohistochemical staining showed that most tumour cells were positive for WT-1 (Figure 7), CD57 (Figure 8), MIB-1, Vimentin, and EMA, while CK7 staining showed weak focal positivity. In two cases, the tumour cells exhibited epithelial elements, lacked the typical architecture of tubules and glomeruloid bodies, and showed atypia and mitotic activity (Figure 9). The proliferation index of the MAs was 3–5% according to the MIB-1 count. The following composite tumours with foci of malignant tumour cells were found in six patients: papillary tumour (n = 3), oncocytic carcinoma (n = 1), adenocarcinoma (n = 1), and chromophobe cell carcinoma (n = 1). The 18 cases were divided into three subtypes according to the pathological findings: classic MA (n = 10), malignant MA (n = 2), and composite MA with coexistence of different malignant components (n = 6). Pathological examination revealed that six (33.3%) tumours had other carcinoma components concomitantly and that two (11.1%) were malignant MA, with a surprisingly high proportion of malignant case. All of these pathological findings indicated the presence of MA subtypes and provided useful information. When stratified by malignant component groups, no significant difference in prognosis was found (p > 0.05).

### Follow-up

All patients were followed up with physical examinations, laboratory tests, chest X-rays, and renal ultrasound or abdominal CT every 3–6 months and then annually. Clinical outcomes were estimated from the date of surgery to the date of death or last follow-up. The median postoperative follow-up period was 56 months (range, 30–86 months), and no local recurrence or metastatic lesions were found with the exception of one patient who developed distant metastasis pathologically diagnosed as malignant MA.

## Discussion

MA was well recognised as a distinct entity in 1988 and was subsequently considered to be a separate entity [3]. The concept of MA has recently been broadened to include MAs, adenofibromas and stromal tumours. There is a female preponderance and a peak age of occurrence in the fifth or sixth decade of life. MA constitutes approximately 0.2% of all adult renal epithelial neoplasms. The incidence of MA in our institution accounts for <1% of all renal tumours, similar to previous reports. Approximately 100 cases of MA have been reported in the English-language literature to date [1]. However, most reports focused mainly on pathology; few reports on the clinical or radiological features are available.

Histogenetically, MA contains renal epithelial or stromal cells. It is postulated to be a benign counterpart of Wilms’ tumour and may be derived from remnants of metanephric blastemal or embryonic renal tissue. MA is considered to represent the most hyperdifferentiated end of the nephroblastoma spectrum and might sometimes coexist with Wilms’ tumour [4]. The genetic profile and chromosomal abnormalities of MA are distinct from those of papillary renal cell carcinoma and Wilms’ tumour. The simultaneous presence of *BRAF* gene mutation and 2p deletion plays a great role in the pathogenesis of MA [5].

Microscopically, the tumour cells have uniformly small and indistinct nucleoli with scanty cytoplasm. Variable proportions of cells proliferate with the formation of small glomeruloid bodies. Immunohistochemical staining shows that most tumour cells are positive for MIB-1, vimentin, EMA, WT-1, and CD57; in contrast, CK7 staining exhibits weak focal positivity. In rare cases, the tumour cells have epithelial elements, lack the typical architecture of tubules and glomeruloid bodies, and show atypia and mitotic activity. Atypical MA needs to be differentiated from Wilms’ tumour, nephrogenic rests, and papillary renal cell carcinoma [6]. MA has morphological similarities to solid papillary renal cell neoplasms; both exhibit significant similarities such as well-circumscribed tumours comprising small tightly packed cells arranged in solid sheets or ill-defined tubules. Some of the morphological features overlap; thus, the differential diagnosis is crucial. MA must also be distinguished from metastatic cancers, particularly those of the thyroid gland and lung. Despite the overlapping features, careful morphological evaluation, especially immunohistochemical staining with CD57, WT1, and CK7, may be useful for differentiation and accurate diagnosis. Meanwhile, genetic analysis may facilitate discrimination in difficult cases. The presence of cytological atypia, mitoses, and anaplastic foci favour the diagnosis of malignant MA, especially distant metastasis. Malignant MA tumours such as metanephric adenocarcinoma, mixed MA, and papillary carcinoma have also been reported [7].

Surprisingly, tumours with typical histological characteristics of MA can present with metastatic disease [2]. Although the natural history of these composite tumours is unknown, they theoretically exhibit aggressive behaviour and the potential for metastasis. In the present study, MA with other concomitant tumour types was determined to be composite MA. This was based on the existing literature stating that a tumour mainly comprising MA that exhibits sporadic concurrent tumours should be classified as a subtype of MA. Composite MA with a co-existing malignant component such as papillary renal cell carcinoma also has metastatic potential [8,9]. The features of these composite tumours are emphasised to promote a better and broader understanding of this uncommon tumour. Notably, oncocytic carcinoma, renal adenocarcinoma, and chromophobe cell carcinoma mixed with MA were reported for the first time in the present investigation. Meanwhile, the cells of two tumours had atypical epithelial elements and mitotic activities, lacked the typical architecture of tubules, and glomeruloid bodies, and were pathologically diagnosed as malignant MA; one tumour was found to be a lung metastasis 46 months postoperatively. The pathological criteria of malignant MA are not well established, and rare metastatic MA has been reported. In contrast to typical MA, malignant MA comprises hypercellular uniform cells in a solid-acini pattern; the cells are variable in size, the nucleoli are prominent, and some cells show increased numbers of mitoses with small uniform nuclei. The diagnosis of malignant MA requires the incorporation of clinical information, histopathological features, and related immunohistochemical staining markers.

Clinically, MA occurs predominantly in adult women and rarely in children,the reported age ranged from 15 months to 83 years [10]. Most patients with MA are asymptomatic or present with nonspecific clinical manifestations such as haematuria, a palpable mass, flank pain, or chyluria [11]. Polycythaemia, which may be associated with para-neoplastic syndrome, is frequently reported among patients with MA. Most patients in the present series were asymptomatic, and no special symptoms were noted. The regular performance of physical examinations has led to a rise in the incidental detection of asymptomatic renal masses. Additionally, MA may be multifocal or bilateral [12,13]. Laboratory tests would be less useful in this setting because no special tumour markers are noted. Urinalysis and renal and hepatic function tests were essentially within normal limits in our series.

Various imaging modalities may be used to characterise MAs. With respect to echogenicity, MA is a hypovascular tumour and has most often been described as a hyperechoic mass [14]. However, the tumours in the present study were hypoechoic, isoechoic, and hyperechoic in nine, four, and five patients, respectively. Abdominal three-phase contrast-enhanced CT was performed in all 18 patients, and no obvious correlations between morphologic features and characteristic CT imaging features were found. No radiological findings were of substantial help in differentiating MA from malignant renal tumours, especially for small masses. In our series, data on the tumour-spreading patterns, lymphadenopathy, and enhancement patterns were recorded and retrospectively analysed. An enlarged lymph node was noted in one patient; the node was pathologically proven to have an inflammatory reaction, similar to a pseudometastatic lesion. The most common CT imaging characteristic was the presence of homogeneous and well-defined solid renal masses (n = 15, 83.3%), and the least common was the presence of heterogeneous or centrally located low-attenuation masses (n = 3, 16.7%). Contrast-enhanced CT revealed hypoattenuating heterogeneous masses with varying degrees of contrast enhancement in 16 (88.9%) patients, while 2 (11.2%) did not show increased attenuation. On unenhanced CT, one tumour (5.6%) showed scattered calcification with higher attenuation than the renal parenchyma. MA appears to be more commonly calcified than other neoplasm [15], which is speculated to be related to the presence of psammomatous calcification or a high nuclear-to-cytoplasmic ratio. Less frequently reported is hypoattenuation or predominantly cystic lesions consistent with necrosis [16]. The hypovascularity of MA seems to reflect the histological findings of mainly acinar and tubular patterns with few vessels. The magnetic resonance imaging features of MA are unspecific; limited cases showed hypointense or isointense lesions on both T1- and T2-weighted magnetic resonance images [17].

Given the rarity of this tumour and lack of pathognomonic clinical and radiographic criteria, pathologic examination is necessary to establish a definitive diagnosis. Because of the undetermined radiological characteristics of MA, several reports recommend percutaneous fine-needle aspiration to confirm the diagnosis preoperatively [18]. However, differentiation of MA from Wilms’ tumour based on fine-needle aspiration biopsy may be difficult [19]. MA may be mixed with other neoplasms that may not be detected by intraoperative biopsy; thus, intraoperative frozen section is not recommended.

From a diagnostic and therapeutic viewpoint, most renal masses should be regarded as malignant and managed surgically; the exception is small renal masses with clinically benign behaviour. Accurate preoperative diagnosis could facilitate optimal management. More widespread recognition of this rare tumour and its subtypes is of great importance for appropriate management of this disease. Our initial classification of three subtypes of MA may contribute to the establishment of guidelines for the management of MA and help in selecting an appropriate surgical method.

Awareness of these subtypes may avoid diagnostic confusion, especially when percutaneous biopsy is indeterminate. When choosing a treatment modality, it might be possible to propose conservative treatment or active surveillance, especially in patients with contraindications to surgery [20]. It should be emphasised that these tumours may not be entirely benign and that their biologic behaviour is uncertain, particularly malignant tumours and those with malignant components. Thus, careful active surveillance may be needed even for MAs of <4 cm. Continued growth or metastatic potential may be lethal; in our opinion, therefore, MA should be routinely resected. Although nephron-sparing surgery is currently the reference standard treatment for clinically localised T1a renal tumours, subjective clinical factors such as surgeon biases and tumour characteristics, including the growth pattern, more likely influence the decision-making process regarding the most appropriate treatment method. Small renal tumours, especially exophytic and peripheral tumours, are ideal candidates for nephron-sparing surgery, either open or laparoscopic partial nephrectomy [21]. Several efficacious therapeutic procedures, such as cryoablation or radiofrequency ablation, are alternative treatment options [22]. In our series, 14 patients underwent radical nephrectomy and only four underwent partial nephrectomy. Most of our patients underwent nephrectomy mainly because of the difficult preoperative differentiation of their lesions from malignant renal tumours. Because imaging is unable to exclude renal cell carcinoma, centrally located or anatomically complex masses should be treated by radical nephrectomy [23].

Patients with MA treated with partial or total nephrectomy have an excellent prognosis. Only one patient in the present series developed distant metastasis 46 months after surgery. Long-term active surveillance is necessary because of the uncertainty of the biological behaviour and potentially composite malignant components of MA. Further studies on various subtypes are needed to identify the possibility or occurrence of metastasis. Metastatic MA containing foci of papillary carcinoma to local lymph nodes were reported in one study; surprisingly, however, the metastatic lesion was an MA, not a papillary carcinoma [7]. Therefore, aggressive intervention is needed for composite MA with a coexisting malignant component.

There were several limitations in the present study. First, the retrospective design and involvement of a single centre might have introduced patient selection bias as well as treatment bias with respect to surgeon preference. Additionally, the time interval of 10 years may have changes in surgical techniques in our institute. Second, there was lack of further molecular analysis of each subtype to elucidate its histogenesis. Third, certain limitations were unavoidable considering the relatively small number of patients and the scarcity of different tumour subtype variants. Moreover, it was not possible to determine the percentage of morphological differentiation in the whole group of specimens. Because only two malignant cases were included in this study, statistical analysis and determination of significant differences were limited.

## Conclusions

We demonstrated multiple variations in MA subtypes, suggesting that their classification spectrum might be wider than originally described. These interesting findings urge timely surgical treatment in all patients with MA. The concept of the disease risk associated with malignant potential has been developed to aid clinicians when deciding on treatment strategies; therefore, regular follow-up is recommended.
